# Incorporating Radiation Oncology into Immunotherapy: proceedings from the ASTRO-SITC-NCI immunotherapy workshop

**DOI:** 10.1186/s40425-018-0317-y

**Published:** 2018-01-29

**Authors:** Ariel E. Marciscano, Joshua M. Walker, Heather M. McGee, Michelle M. Kim, Charles A. Kunos, Arta M. Monjazeb, Stephen L. Shiao, Phuoc T. Tran, Mansoor M. Ahmed

**Affiliations:** 1 Department of Radiation Oncology & Molecular Radiation Sciences, The Sidney Kimmel Comprehensive Cancer Center, Johns Hopkins University School of Medicine, 1550 Orleans Street CRB2, RM 406, Baltimore, MD 21231 USA; 2 Department of Radiation Medicine, Oregon Health & Science University, Portland, OR USA; 3 Department of Radiation Oncology, Icahn School of Medicine at Mount Sinai, New York, NY USA; 4 Department of Radiation Oncology, University of Michigan, Ann Arbor, MI USA; 5 Investigational Drug Branch, Cancer Therapy Evaluation Program, National Cancer Institute, Bethesda, MD USA; 6 Department of Radiation Oncology, UC Davis Comprehensive Cancer Center, Sacramento, CA USA; 7 Department of Radiation Oncology, Cedars-Sinai Medical Center, Samuel Oschin Comprehensive Cancer Institute, Cedars-Sinai Medical Center, Los Angeles, CA USA; 8 Radiation Research Program, National Cancer Institute, Bethesda, MD USA; 9 Molecular Radiation Therapeutics, Radiation Research Program, National Cancer Institute, National Institutes of Health, 9609 Medical Center Drive, Rockville, MD 20892-9760 USA

**Keywords:** Radiation oncology, Radiation therapy, Radiotherapy, Immunotherapy, Immune checkpoint blockade, Immune checkpoint inhibitors, ASTRO, SITC, Combinations, Cancer

## Abstract

Radiotherapy (RT) has been a fundamental component of the anti-cancer armamentarium for over a century. Approximately half of all cancer patients are treated with radiotherapy during their disease course. Over the two past decades, there has been a growing body of preclinical evidence supporting the immunomodulatory effects of radiotherapy, particularly when combined with immunotherapy, but only anecdotal clinical examples existed until recently. The renaissance of immunotherapy and the recent U.S. Food and Drug Administration (FDA) approval of several immune checkpoint inhibitors (ICIs) and other immuno-oncology (IO) agents in multiple cancers provides the opportunity to investigate how localized radiotherapy can induce systemic immune responses. Early clinical experiences have demonstrated feasibility of this approach but additional preclinical and clinical investigation is needed to understand how RT and immunotherapy can be optimally combined.

To address questions that are critical to successful incorporation of radiation oncology into immunotherapy, the American Society for Radiation Oncology (ASTRO), the Society for Immunotherapy of Cancer (SITC) and the National Cancer Institute (NCI) organized a collaborative scientific workshop, Incorporating Radiation Oncology into Immunotherapy, that convened on June 15 and 16 of 2017 at the Natcher Building, NIH Campus in Bethesda, Maryland. This report summarizes key data and highlights from each session.

## Radiation and immunotherapy: a promising partnership?

Radiotherapy (RT) has been a fundamental component of the anti-cancer armamentarium for over a century. Approximately half of all cancer patients are treated with radiotherapy during their disease course [1]. Over the two past decades, there has been a growing body of preclinical evidence [2–4] supports the immunomodulatory effects of radiotherapy, particularly when combined with immunotherapy, but only anecdotal clinical examples existed until recently [5–7]. The renaissance of immunotherapy and the recent Food and Drug Administration (FDA) approval of several immune checkpoint inhibitors (ICIs) and other immuno-oncology (IO) agents in multiple cancers provides the opportunity to investigate how localized radiotherapy can induce systemic immune responses. Early clinical experiences have demonstrated feasibility of this approach but additional preclinical and clinical investigation is needed to understand how RT and immunotherapy can be optimally combined [8–12].

To address questions that are critical to successful incorporation of radiation oncology into immunotherapy, the American Society for Radiation Oncology (ASTRO), the Society for Immunotherapy of Cancer (SITC) and the National Cancer Institute (NCI) organized a collaborative scientific workshop that convened on June 15 and 16 of 2017 at the Natcher Building, NIH Campus in Bethesda, Maryland.

Scientific sessions and breakout discussions were held to foster discussion and collaboration with a focus on the following key issues:

https://www.astro.org/Meetings-and-Education/ASTRO-Meetings/2017/Immunotherapy-Workshop/2017-Immunotherapy-Workshop/:

1. Mechanisms of immune stimulation by radiotherapy and other local therapies
2. Potential biomarkers for radiotherapy and immunotherapy
3. Development of rational combinations of radiotherapy and immunotherapy

## Local therapies and immune stimulation

The first session of the workshop reviewed the background and rationale for combining local therapies with immunotherapy. After the first keynote speech by Dr. Silvia Formenti (Weill Cornell Medicine), the first session of the workshop included talks by Dr. Sandra Demaria (Weill Cornell Medicine), Dr. Phouc Tran (Johns Hopkins Medicine), Dr. Chandan Guha (Albert Einstein College of Medicine), and Dr. Andrea Facciabene (University of Pennsylvania) on local therapies and systemic immune stimulation.

Although, the combined effects of RT and immunotherapy have been increasingly well characterized in small animal models, the optimal radiation dose and fractionation and the mechanism of synergy with immunotherapy remains to be firmly established. The keynote introduction by Dr. Silvia Formenti addressed this important issue and presented recent work performed in conjunction with Dr. Sandra Demaria and their team at Weill Cornell Medicine. These investigators previously demonstrated that hypofractionated RT can more effectively stimulate abscopal responses than high-dose single fraction RT when combined with cytotoxic T-lymphocyte antigen-4 (CTLA-4) ICIs [13]. Following up on this important finding, this group and others have now elucidated a possible mechanism with the recognition that Type-I interferon (IFN) signaling originating from irradiated tumor cells is critical for recruitment and activation of BATF3-dependent dendritic cells (DCs) to the tumor and subsequent RT-driven anti-tumor immunity [14, 15].

Vanpouille-Box and colleagues observed increased double-stranded-DNA (dsDNA) accumulation and c-GAS/STING (STimulator of INterferon Genes) activation within the cytosol of cells irradiated with 8 Gray (Gy) × 3 compared to 20 Gy × 1. This led to their discovery that radiation in doses greater than ~ 10–12 Gy per fraction induces expression of the (3′- > 5′) three prime repair exonuclease 1, TREX1, which degrades cytosolic dsDNA. The dose-dependent induction of TREX1 appears to abrogate radiation-induced c-GAS/STING activation of Type I IFN responses and dampen systemic anti-tumor immunity [15]. In the context of CTLA-4 blockade, hypofractionated regimens (i.e. 8 Gy × 3) result in more robust CD8+ T-cell effector function in response to tumor antigen, regression of non-irradiated tumors or “abscopal” lesions, and increased survival when compared to a high-dose single fraction approach (20 Gy × 1). This interesting work has helped to improve our understanding of the mechanisms underlying the immunogenic effects of hypofractionated RT, and how to best combine RT with immunotherapy.

Dr. Chandan Guha of Albert Einstein College of Medicine provided an alternative perspective suggesting that ablative local therapies including high-dose single-fraction RT can also effectively potentiate systemic immune responses. Indeed, using a metastatic murine lung carcinoma model, his group had previously demonstrated that locally ablative doses (60 Gy) in combination with Flt-3 ligand resulted in cure and long-term protective immunity in a subset of mice [3, 16]. Dr. Guha next shared the results of his interesting preclinical work focusing on locally-directed ultrasound therapy to generate an in situ tumor vaccine. The work focused on the potential to use low-intensity focused ultrasound (LOFU) to initiate an unfolded protein cellular stress response characterized by upregulated heat shock proteins and denatured proteins which effectively conditions tumor cells to generate systemic immune responses in response to subsequent locally ablative therapy [17]. Dr. Guha described that ablative approaches, including high-intensity focused ultrasound (HIFU) and stereotactic ablative RT, may be optimal partners to generate T-cell mediated systemic immunomodulation initiated by LOFU [18].

On a similar note, Dr. Phuoc T. Tran of Johns Hopkins Medicine also addressed the question of whether other ablative modalities can result in similar systemic immune stimulation and abscopal responses. Alternative ablative strategies include cryotherapy, radiofrequency ablation, and HIFU [19]. There are published results using each of these techniques in conjunction with immunotherapy that suggest the ability to augment anti-tumor immunity [20–22]. However, there is little published data directly comparing ablative techniques, and their relative capacity to synergize with systemic immunotherapy is unknown. Dr. Tran presented preliminary data from mice undergoing RT, cautery, or cryotherapy in combination with ICIs, demonstrating the development of systemic increases in CD8+ T-cell populations following all three treatment modalities.

Furthermore, Dr. Tran provided his insights on the ongoing challenges and new opportunities for immunotherapy in prostate cancer and other poorly immunogenic “cold” tumors. The recent failed phase III CA184-043 trial comparing the addition of ipilimumab (anti-CTLA-4) to palliative bone-directed radiotherapy (8 Gy × 1) in heavily pre-treated metastatic castrate-resistant prostate cancer patients did not improve overall survival but did provide a progression-free survival benefit. Further, a subset of patients with low burden, non-visceral metastatic disease derived a survival advantage with the addition of ipilimumab to their treatment regimen [23]. Given this result and the success of Sipuleucel-T in minimally symptomatic/asymptomatic metastatic prostate cancer it might be suggested that radiation combined with immunotherapy might be more effective among men with less advanced disease [24]. To this end, Dr. Tran discussed his group’s ongoing randomized phase II ORIOLE trial in men with oligometastatic hormone-sensitive disease evaluating if the application of stereotactic ablative RT can alter the natural history of this disease and delay progression relative to observation [25]. This study incorporates robust immunologic correlates which may provide insights on opportunities for synergy with IO agents in prostate cancer.

The final presentation during this session was by Dr. Andrea Facciabene from the University of Pennsylvania, who presented intriguing preclinical data on the potential for antibiotic therapy directed at gram-positive gut microbiota (vancomycin) to augment the anti-tumor effects of RT in three different murine xenograft models of melanoma, cervical carcinoma and lung carcinoma. Indeed, these enhanced anti-tumor immune effects observed with combining vancomycin with RT were mediated by IFNγ-expressing CD8+ T-cells. Further, the addition of vancomycin enhanced the frequency of CD103+ DCs capable of cross-presenting tumor antigen. These preliminary data carry additional importance given that several recent high-impact studies have demonstrated that the efficacy of immunotherapy is dependent on the intestinal microbiota [26–28]. Indeed, the gut microbiome is rapidly becoming an important clinical biomarker and therapeutic target for cancer immunotherapy [29, 30].

## Biomarkers in immunotherapy and radiation oncology

As we integrate radiation and immunotherapy combinations into clinical practice, one of the greatest challenges and opportunities will be the development and integration of biomarkers to predict which patients will respond to these therapies. During the second session of the workshop, Dr. Lisa Butterfield (University of Pittsburgh Medical Center), Dr. Timothy Chan (Memorial Sloan Kettering Cancer Center), Dr. Maximillian Diehn (Stanford University), Dr. Sridhar Nimmagadda (Johns Hopkins Medicine), and Dr. Heather McGee (Icahn School of Medicine at Mount Sinai) presented their work to identify biomarkers of response to immunotherapy and/or RT.

This session began with a comprehensive overview by Lisa Butterfield on the SITC Biomarkers Task Force and its four working groups focusing on: 1) immune monitoring standardization and validation, 2) new developments in assays and technologies, 3) high throughput approaches for assessment of immune regulation and modulation, and 4) baseline immunity in the tumor microenvironment (TME) and outcome prediction [31]. Dr. Butterfield highlighted the vast array of emerging technologies and high-throughput approaches, such as next-generation sequencing for mutational load/neoantigen assessment, mass cytometry (CyTOF), TCR sequencing, Nanostring, and multiplex immunofluorescence. While these technologies allow for a vast array of questions to be addressed with limited tissue specimens, the quantity and complexity of data produced will require advanced bioinformatics to interpret these different data sets, as well as clinical expertise to identify the most clinically relevant information. An additional issue raised by Dr. Butterfield is the lack of standardized conditions for the collection and storage of tissue specimens and lack of pre-analytical validation and guidelines for peripheral blood mononuclear cells (PBMC) processing. Furthermore, she mentioned the need to focus on immune cell composition within the TME to try to develop surrogates for measuring these cell types in PBMC. This will require coordinated collection of PBMC and tissue specimens at various timepoints before, during and after immunotherapy to understand temporal relationships and interactions between the local and the systemic immune response, as well as to assess mechanisms of resistance. Dr. Butterfield emphasized that several of the issues encountered with the biomarker development and validation for IO agent monotherapy are also likely to be relevant and applicable to biomarkers for RT-IO combinations.

Dr. Timothy Chan from Memorial Sloan Kettering Cancer Center discussed molecular and genomic determinants of response to immunotherapy which will help guide rational combination with RT. Dr. Chan’s laboratory performed whole-exome sequencing on two cohorts of non-small cell lung cancer patients treated with pembrolizumab (anti-PD-1) and uncovered an important link between tumor mutation load and response to ICIs [32, 33]. Specifically, high non-synonymous somatic mutational load correlated with treatment efficacy and the development of neoantigen-specific CD8+ T-cell responses paralleled tumor regression in a patient with favorable response to ICI. This reinforced the concept that high neoantigen burden influences the response to programmed cell death protein-1 (PD-1) blockade and that CD8+ T cells targeting tumor-specific neoantigens are drivers of therapeutic anti-tumor immunity [33].

An additional promising avenue to non-invasively monitor and evaluate response to RT and immunotherapy is the emerging field of circulating tumor DNA (ctDNA) profiling, which was discussed by Dr. Maximillian Diehn from Stanford University [34]. Dr. Diehn’s group has developed and implemented CAncer Personalized Profiling by deep Sequencing (CAPP-Seq), a novel next-generation sequencing-based approach for ultrasensitive ctDNA detection [35]. Applications of this CAPP-Seq technology range from early detection to non-invasive tumor genotyping of targetable mutations, as well as detection of minimal residual disease or early disease recurrence. One of the most unique aspects of this technology is that it may help us evaluate “real-time Darwinian evolution of tumor clones” and mechanisms of drug resistance without the need for repeat tumor biopsies. Clinical studies are currently underway to validate this concept in several tumor types [25, 36]. CAPP-Seq and ctDNA quantification could become a powerful assay and biomarker to help individualize treatment for patients undergoing RT or combined therapy with immunotherapy.

The next presentation also addressed the importance of developing peripheral blood-based biomarkers to predict which patients are most likely to respond to treatment. Dr. Heather McGee of Mount Sinai, presented work done in collaboration with Drs. Arta Monjazeb and Megan Daly, from the University of California Davis, to address this unanswered question. They designed a prospective blood collection study to evaluate the immune response to stereotactic ablative RT as a monotherapy and to determine how the immune response differs based on the site that is irradiated. They observed a decrease in circulating cytotoxic natural killer (NK) cells after stereotactic ablative RT to parenchymal sites, but not after irradiation of bone or brain. They also observed an increase in TIM-3+ NK cells and an increase in activated memory CD4+ and CD8+ T-cell subsets after stereotactic ablative RT to the parenchymal sites which was not observed with RT directed to non-parenchymal sites. Future studies correlating these immune markers with clinical outcomes will determine if any of these immune changes can be used as biomarkers for immune responses to RT. This data emphasizes the importance of RT target/site in future clinical trials combining radiation with immunotherapy.

Novel imaging modalities may be well-suited to serve as biomarkers for IO agents and have distinct advantages over conventional methods such as immunohistochemistry (IHC) or computed tomography (CT) imaging. Within the field of molecular oncologic imaging, immuno-PET, a positron emission tomography (PET)-based imaging technique that utilizes radiolabeled monoclonal antibodies against targets of interest is being actively developed to assay/image programmed cell death-ligand 1 (PD-L1) expression and other immune targets [37–39]. Dr. Sridhar Nimmagadda of Johns Hopkins Medicine presented his intriguing preclinical work demonstrating the ability to non-invasively and comprehensively monitor PD-L1 expression in spatial and temporal dimensions using a copper-64 labeled peptide with the hopes of translating this technology to the clinic in the near future [40]. Whole-body molecular imaging may help circumvent issues associated with sampling error or tissue heterogeneity.

While the compendium of sophisticated tools and cutting-edge technologies (i.e. CyTOF, multiplex IHC, whole exome and TCR sequencing, CAPP-Seq, molecular imaging, CAPP-Seq and multiparametric flow cytometry) for interrogating the immune response continues to expand, the major challenge is to determine how we can use this abundance of information to identify and validate biomarkers that correlate with clinically meaningful outcomes.

## Rational combinations of radiation and immunotherapy

During the final session of the workshop, Drs. Marka Crittenden (Earle A. Chiles Research Institute), Michael Lim (Johns Hopkins Medicine), Andy J. Minn (University of Pennsylvania) and Todd Aguilera (Stanford University) presented their most recent work examining different combinations of RT and immunotherapy. While tremendous strides have been made with respect to optimal dose-fractionation, as highlighted during Dr. Silvia Formenti’s keynote address, there is still much work to be done to successfully integrate RT and immunotherapy.

A critical aspect of combination approaches is the relative timing and sequencing of treatments, specifically whether RT is more efficacious when delivered before, after, or concurrently with immunotherapy. Dr. Marka Crittenden of Earle A. Chiles Research Institute reviewed preclinical work that suggests the timing of immunotherapy relative to RT is critical. In a series of experiments combining hypofractionated RT with either CTLA-4 blockade or OX40 agonists, Young and colleagues reported that administration of CTLA-4 blockade before RT was superior to alternative schedules because this strategy permitted depletion of immune-inhibitory regulatory T cells which restrain CD8+ effector function and anti-tumor immunity [41]. Alternatively, treatment with OX40 agonists after RT yielded the most robust responses compared to other sequencing because RT induces transient expression of OX40 on CD8+ T cells and therefore promotes efficient T-cell co-stimulation [41]. These findings underscore the importance of understanding the mechanism of action of IO agents when considering the optimal timing or sequencing with radiation.

The importance of immunologically rigorous preclinical tumor models was emphasized by Dr. Crittenden as transplantable tumor models induce immune responses upon tumor implantation. Indeed, provocative data using a series of experiments preventing T cell priming at the time of tumor implantation suggest that much of the response to immunotherapy and radiation/immunotherapy combinations observed in murine models may be dependent upon T-cell activation at the time of tumor challenge. This notion is further supported by work presented by Dr. Yvonne Mowery of Duke University utilizing a genetically engineered mouse model of sarcoma with a conditional p53 knockout [42]. Interestingly, tumors implanted as subcutaneous allografts were cured in roughly 80% of cases with anti-PD-1 therapy alone. However, tumors that developed in response to conditional knockout of p53 had minimal response to ICIs. This data is consistent with others demonstrating that if anti-tumor immune responses develop in the immunosuppressive TME and experience chronic antigen stimulation they become terminally and functionally exhausted [43–45].

Dr. Michael Lim shared his recent work on the immunological implications of systemic chemotherapy and potential ways to mitigate systemic immune suppression in an effort to enhance synergy with ICIs for brain tumors. Using an intracranial orthotopic glioma model, Mathios and colleagues described that administration of systemic chemotherapy resulted in profound lymphopenia and deleterious effects upon synergy with anti-PD-1 ICIs [46]. The administration of local chemotherapy, via implantation of chemotherapy-covered polymers akin to Gliadel® wafers, did not blunt peripheral immune composition. Intriguingly, in combination with anti-PD-1, local chemotherapy enhanced antigen presentation via intratumoral DCs which lead to superior long-term survival and immunological memory formation relative to systemic chemotherapy. These findings yield important observations regarding the immune-potentiating potential and optimal application of local therapies, including RT. Supporting this point, Dr. Lim’s group at Johns Hopkins Medicine has demonstrated impressive synergy in similar glioma models combining stereotactic RT with anti-PD-1 and various other IO agents [47, 48].

This session also touched upon the importance of tumor heterogeneity, differences in cancer-immune phenotype and challenges ahead for poorly immunogenic tumors [49]. The concept of “hot/inflamed” tumors, characterized by T-cell infiltration and a type I IFN signature, versus “cold/non-inflamed” tumors highlighted the need for novel IO strategies to address “cold” tumors that fail to respond to ICIs, RT, or their combination [50]. Dr. Todd Aguilera of Stanford University eloquently described the role of Axl expression as a mechanism of tumor immune evasion and its relationship to radiosensitivity and synergy with immunotherapy [51]. Using a transgenic PyMT breast cancer murine model, comprehensive profiling of two tumor clones with differential response to immune checkpoint inhibition demonstrated significant upregulation of Axl among resistant tumors. Genetic deletion of Axl restored sensitivity to immunotherapy and permitted robust CD8+ T-cell infiltration, enhanced major histocompatibility complex (MHC) class I presentation and also decreased skewing toward the immune-suppressive M2-macrophage phenotype. These data highlight critical determinants of anti-tumor immunity in response to radiation and suggest the potential of targeting the Axl pathway to promote synergy with RT. Interestingly, Crittenden’s group has also demonstrated that Mertk, a TAM family of receptor tyrosine kinases (of which Axl belongs), is significantly upregulated on tumor-associated macrophages following hypofractionated RT. Inhibition of Mertk in combination with TGFβ blockade might effectively boost anti-tumor immunity and circumvent radiation-mediated immunosuppression in poorly immunogenic tumors [52].

Finally, Dr. Andy J. Minn of the University of Pennsylvania described a fascinating body of work that highlighted the immense complexity in deciphering mechanisms of response and acquired resistance to immunotherapy and RT combinations. While the ability of radiation to drive inflammation via STING/Type I IFN pathway activation is increasingly appreciated as an important mediator of anti-tumor immunity, Dr. Minn’s group has provided evidence that persistent IFN signaling also carries deleterious effects [15, 53]. Initial work from his group sought to understand treatment failures in a clinical trial of hypofractionated RT in combination CTLA-4 blockade in melanoma. Preclinical work by Twyman-Saint Victor et al. demonstrated that IFN-driven upregulation of PD-L1 on tumor cells is an important mechanism of immune evasion [54]. The addition of PD-1/PD-L1 blockade (dual immune checkpoint inhibition and radiation) to RT/anti-CTLA-4 overcame PD-L1 adaptive immune resistance in this model. Nevertheless, high proportions of patients fail to respond to PD-1/PD-L1 blockade while other patients develop acquired resistance or late relapses [55, 56]. As such, Dr. Minn’s group elucidated a complex immune resistance program that is initiated through prolonged IFN signaling which blunts response to checkpoint blockade and represents a PD-L1-independent mechanism of adaptive resistance [53]. The IFN-driven immune resistance program is characterized by epigenomic modulation of immunosuppressive IFN-stimulated genes leading to expression of various T-cell immune-inhibitory receptors, chronic T-cell exhaustion and ultimately, treatment failure. Interestingly, Janus kinase (JAK) inhibitors suppressing tumor IFN signaling might represent a pharmacologic strategy that may help overcome this multigenic resistance program [53]. Moving forward, improving our mechanistic understanding of how radiation can promote the immunogenic effects of IFN signaling while preventing immune resistance will be of interest.

Effective anti-tumor immunity is counter-balanced by various mechanisms of immune tolerance and immune suppression that are intrinsic to the TME and/or induced by RT [57]. When combined with RT, immunotherapies that target immune evasion or resistance may help tilt the balance towards effective anti-tumor immune responses by limiting radiation-mediated immunosuppressive effects and harnessing the immunogenic potential of RT. To enhance treatment responses, immunotherapeutic approaches must be tailored to the specific host and TME immune status. Importantly, the diverse effects of radiation upon host and tumor immunity suggests multiple pathways to synergize with different types of IO agents [58].

## Conclusions and future directions

Understanding molecular, genetic and immunologic mechanisms that dictate the immunomodulatory effects of radiotherapy will be critical to successfully combine radiation and immunotherapy. Important progress is being made in this arena but robust clinical evidence is lacking [14, 59]. Key outstanding questions for the field of radiation oncology pertain to dose/fractionation, sequencing with immunotherapy and radiation target site/volume. Important themes arising from scientific breakout sessions have highlighted future directions for this field:

- Local Therapies and Immune Stimulation
  - The mechanisms of RT-mediated immunogenicity are now being elucidated, with recent evidence demonstrating that one important pathway is dsDNA breaks generated by hypofractionated RT regimens stimulating Type I IFN expression through activation of the c-GAS/STING pathway.
  - While the most robust data exists studying the combination of RT and ICIs, there is also evidence that other ablative modalities (i.e. HIFU) may effectively stimulate T-cell immunity and synergize with RT.
  - There is increasing data that RT may synergize with IO agents in “cold” or poorly immunogenic histologies previously believed to derive modest benefit from immunotherapy as a monotherapy.
- Biomarkers in Immunotherapy and Radiation Oncology
  - Biomarker discovery for combination regimens can guide appropriate patient selection, assist in therapeutic decision making, and improve assessment of treatment response.
  - A variety of specimens can be collected, including urine for metabolic biomarkers, stool for microbiome biomarkers, PBMC as well as tissue biopsies at the tumor site.
  - New cutting-edge technologies, such as next-generation sequencing for assessment non-synonymous somatic mutational load, CAPP-Seq for ctDNA quantification, and Immuno-PET, are being developed and verified in patient populations.
  - These novel techniques and tools will help us monitor and assay immunological and biological responses to radiation and immunotherapy, but the challenge is to identify and validate biomarkers that correlate with clinically meaningful outcomes.
- Rational Combinations of Radiation and Immunotherapy
  - Patient and tumor-specific factors as well as the mechanism of action of each IO agent are important.
  - Optimal dose/fractionation and sequence of therapies may not be generalizable to all radiation-immunotherapy combinations.
  - Looking beyond immune checkpoint inhibitors as a monotherapy – combining radiation with multi-agent IO regimens that target different aspects of tumor-immunity: (1) stimulate de novo immune responses, (2) promote immune effector cell function and (3) reverse T-cell dysfunction or overcome immune suppression.

Thoughtful and collaborative clinical trial design is paramount to answer these questions definitively and to validate current understanding regarding radiation-mediated in situ vaccination. As a community, a collective effort should be placed on integrating radio-immuno-oncology and precision medicine so that novel treatment approaches can be designed based on tumor, immune, environmental and patient-specific factors [60].

## Abbreviations

ASTRO: American Society for Radiation Oncology
CAPP-Seq: Cancer personalized profiling by deep sequencing
CT: Computed tomography
ctDNA: circulating tumor DNA
CTLA-4: Cytotoxic T-lymphocyte antigen-4
DC: Dendritic cell
dsDNA: double-stranded-DNA
FDA: Food and Drug Administration
Gy: Gray
HIFU: High-intensity focused ultrasound
ICI: Immune checkpoint inhibitor
IFN: Interferon
IHC: Immunohistochemistry
IO: Immuno-oncology
JAK: Janus kinase
LOFU: Low-intensity focused ultrasound
MHC: Major histocompatibility complex
NCI: National Cancer Institute
NK: Natural killer
PBMC: Peripheral blood mononuclear cells
PD-1: Programmed cell death protein-1
PD-L1: Programmed cell death-ligand 1
PET: Positron emission tomography
RT: Radiotherapy
SITC: Society for Immunotherapy of Cancer
STING: Stimulator of interferon genes
TME: Tumor microenvironment
